# Prevalence Of Food Insecurity And Associated Factors Among Adults Living With Chronic Pain In Quebec, Canada: A Cross-Sectional Study

**DOI:** 10.1080/24740527.2026.2680295

**Published:** 2026-07-09

**Authors:** Éloïse Farand, Paul Farand, Alexandra Martel, Alex Dodier, Valérie St-Pierre, Anaïs Lacasse, on behalf of the CEMPUS Cohort Research Group

**Affiliations:** a Département de médecine, Faculté de médecine et des sciences de la santé, Université de Sherbrooke; Centre de recherche clinique du Centre intégré universitaire de santé et de services sociaux de l’Estrie - Centre hospitalier universitaire de Sherbrooke, Sherbrooke, Québec, Canada; b Service de cardiologie, Département de médecine, Faculté de médecine et des sciences de la santé, Université de Sherbrooke; Centre de recherche clinique du Centre intégré universitaire de santé et de services sociaux de l’Estrie - Centre hospitalier universitaire de Sherbrooke, Sherbrooke, Québec, Canada; c Département de médecine, Faculté de médecine et des sciences de la santé, Université de Sherbrooke, Sherbrooke, Québec, Canada; d Département des sciences de la santé, Université du Québec en Abitibi-Témiscamingue, Rouyn-Noranda, Québec, Canada

**Keywords:** Associated factors, chronic pain, food insecurity, health equity, prevalence, socioeconomic status, social determinants of health

## Abstract

**Background:**

Food insecurity is the uncertain, insufficient, or inadequate access to, availability of, or utilization of food due to limited financial resources. Research on food insecurity among individuals living with chronic pain is limited.

**Aims:**

Describe the prevalence of food insecurity in this population and to identify associated factors.

**Methods:**

This cross-sectional study used data from 1549 participants from the CEMPUS Cohort living with chronic pain (Quebec, Canada). Participants completed a phone or online questionnaire in 2024. Food insecurity was assessed using the validated Canadian 10-item adult Household Food Security Survey Module. Multivariable logistic regression identified the associated factors.

**Results:**

Food insecurity was reported by 21.4% of the participants aged 18–24, 28.8% of those aged 25–34, 20.4% of those aged 35–44, 17.1% of those aged 45–54, 9.3% of those aged 55–64, and 4.0% of the adults aged 65 and older; prevalence was similar among females and males. Anxiety (OR: 1.125, 95%CI: 1.051–1.205), depression (OR: 1.081, 95%CI: 1.004–1.164), smoking (OR: 2.098, 95%CI: 1.234–3.566), presence of children in the household (OR: 1.927, 95%CI: 1.114–3.333), and pain duration (1–4 years vs. 3–11 months OR: 2.086, 95%CI: 1.025–4.246) were associated with higher odds of food insecurity. Older age and higher household income were associated with lower odds.

**Conclusions:**

While several factors may be amenable to intervention, the observed associations are not necessarily causal, and some factors cannot be directly modified. Nevertheless, our findings from a Quebec sample emphasize the need to consider food insecurity in equity-oriented chronic pain research and the development of supportive strategies.

## RÉSUMÉ

**Contexte :** L’insécurité alimentaire désigne une situation où l’accès à la nourriture, la disponibilité des aliments ou leur utilisation sont incertains, insuffisants ou inadéquats en raison de ressources financières limitées. Les recherches portant sur l’insécurité alimentaire chez les personnes vivant avec une douleur chronique demeurent limitées.

**Buts :** Décrire la prévalence de l’insécurité alimentaire dans cette population et déterminer les facteurs qui y sont associés.

**Méthodes :** Cette étude transversale repose sur les données de 1 549 participants de la cohorte CEMPUS vivant avec une douleur chronique au Québec, Canada. Les participants ont rempli un questionnaire par téléphone ou en ligne en 2024. L’insécurité alimentaire a été évaluée au moyen du module canadien validé d’enquête sur la sécurité alimentaire des ménages pour adultes, qui comporte 10 questions. Les facteurs associés ont été déterminés à l’aide d’une régression logistique multivariée.

**Résultats :** L’insécurité alimentaire a été rapportée par 21,4 % des participants âgés de 18 à 24 ans, 28,8 % de ceux âgés de 25 à 34 ans, 20,4 % de ceux âgés de 35 à 44 ans, 17,1 % de ceux âgés de 45 à 54 ans, 9,3 % de ceux âgés de 55 à 64 ans et 4,0 % des adultes âgés de 65 ans et plus; la prévalence était similaire chez les femmes et les hommes. L’anxiété (RC : 1,125; IC à 95 % : 1,051-1,205), la dépression (RC : 1,081; IC à 95 % : 1,004-1,164), le tabagisme (RC : 2,098; IC à 95 % : 1,234-3,566), la présence d’enfants dans le ménage (RC : 1,927; IC à 95 % : 1,114-3,333) et une durée de la douleur de 1 à 4 ans, comparativement à 3 à 11 mois (RC : 2,086; IC à 95 % : 1,025-4,246), étaient associés à une probabilité plus élevée d’insécurité alimentaire. Un âge plus avancé et un revenu du ménage plus élevé étaient associés à une probabilité plus faible.

**Conclusions :** Bien que plusieurs facteurs puissent se prêter à une intervention, les associations observées ne sont pas nécessairement causales, et certains facteurs ne peuvent être directement modifiés. Néanmoins, nos résultats, tirés d’un échantillon québécois, soulignent la nécessité de tenir compte de l’insécurité alimentaire dans la recherche sur la douleur chronique axée sur l’équité ainsi que dans l’élaboration de stratégies de soutien.

## Introduction

Chronic pain is defined as pain that persists or recurs for longer than 3 months.^1^ While it was long regarded as a symptom, it is now recognized as a distinct disease by the World Health Organization.^1^ Chronic pain affects one in five people^2,3^ and is associated with reduced quality of life, decreased productivity and functioning, social isolation and psychological distress, such as depression and a higher suicide risk.^2,4,5^ Chronic pain is also a major economic burden for the healthcare system, as well as for those affected by the disease and their families. In 2019, the annual direct and indirect costs of chronic pain in Canada were estimated at 40 billion dollars.^2^ The management of chronic pain remains suboptimal to this day. The limited effectiveness of available medications, insufficient training of healthcare professionals, stigma, and lack of equitable, consistent, and timely access to a continuum of care and support all contribute to this situation.^2^

Multiple biological, psychological, and social factors influence the development and perception of pain.^2^ Therefore, certain groups are at higher risk of chronic pain, such as women, seniors, veterans, ethnic minorities, and Indigenous populations.^2,5,6^ Chronic pain is also more prevalent among socioeconomically disadvantaged individuals.^5–9^ For example, it can lead to job loss and economic inactivity.^10,11^ Also, numerous treatments are attempted for pain management that are not covered by insurance which can result in a significant outofpocket financial burden.^12^ Although income and education are commonly used to determine socioeconomic status, food insecurity also reflects financial hardship and its consequences on health suggest it can be an important social determinant of health.^13^ Food insecurity can be defined as uncertain, insufficient, or inadequate access to, availability of, or utilization of food due to limited financial resources, as well as the compromised eating patterns and food consumption that may result.^14^ The prevalence of food insecurity in Canada has considerably increased in the last few years.^15^ In 2021, 15.7% of the people in the ten Canadian provinces lived in a food-insecure household.^15^ After rising for three consecutive years, this proportion reached 25.5% in 2024.^15^ In the province of Quebec, this proportion was 19.8% in 2024.^15^ Contrary to what one might think, food insecurity does not only affect people living in poverty. Most Canadian families (78%) experiencing food insecurity live above the poverty line.^16^ While causal evidence remains limited, recent increases in food insecurity have coincided with substantial increases in the cost-of-living in Canada, including marked increases in food and housing prices.^17,18^

In spite of food insecurity being a risk factor for numerous health issues, such as mental health problems and chronic diseases,^13,19–24^ and becoming more widespread,^15^ few studies have paid attention to the relationship between this issue and chronic pain. A Canadian study published in 2021 demonstrated that food insecure individuals had greater odds of experiencing chronic pain than their food secure peers, in a dose–response fashion and when adjusting for confounders.^25^ A U.S. study from 2023 had similar results.^26^ The association between chronic pain and food insecurity was not only independent of other socioeconomic factors, such as education and income, but outweighed these factors^25,26^ and other established sociodemographic determinants of health, such as sex and ethnicity.^25^ Despite evidence from these comparative studies indicating that individuals with and without chronic pain do not experience food insecurity to the same extent, a gap remains in understanding how food insecurity is distributed and experienced *within* the chronic pain population. In particular, factors associated with food insecurity in this population, including pain-related characteristics, remain poorly understood. Also, food insecurity remains largely unaddressed in chronic pain action plans and strategies, which nonetheless acknowledge broader socioeconomic vulnerabilities, such as poverty, and despite its prominence as a key indicator in national Statistics Canada monitoring, underscoring the need for population-specific evidence among individuals living with chronic pain.

The objectives of this study were to: (1) Describe the prevalence of food insecurity among people living with chronic pain in Quebec, Canada and (2) identify the factors associated with food insecurity among this population. We hypothesize that the likelihood of food insecurity is associated with specific pain-related characteristics, in addition to sociodemographic and clinical factors.

## Methods

The reporting of this manuscript adheres to the Strengthening the Reporting of Observational Studies in Epidemiology (STROBE) statement for cross-sectional studies.^27^

### Study design and data source

This cross-sectional study used data from the 2024 cycle of the “*Cohort as Part of the Undergraduate Medical Studies at the University of Sherbrooke,”* also called the CEMPUS cohort.^28^ This annual survey was created in 2019 to support the academic training of medical students and improve their knowledge on factors that impact the health of the Canadian population. It was described elsewhere.^29^ Most of its content is inspired by Statistics from Canada‘s Canadian Community Health Survey (CCHS) questions.^30^ In the context of the 2024 CEMPUS cycle, data was collected from August 25th to September 25th 2024 from 2887 participants. Participants had to reside in Quebec (Canada) and be able to complete the questionnaire in French or in English to be eligible. Participants were recruited from: (1) respondents of the most recent *Enquête de la santé populationnelle estrienne* (ESPE), a population health survey conducted in the administrative region of Estrie (Quebec, Canada), who had previously consented to be recontacted for research, and (2) participants from previous CEMPUS cycles (permission to be recontacted for participation in the following year’s CEMPUS Cohort is always obtained at the end of the questionnaire).

Third-year medical students at the Université de Sherbrooke were responsible for developing the questionnaire and collecting the data. Interviewers who had received standardized training contacted potential participants by telephone, read the information required to allow free and informed consent, and determined participants’ preferred mode of questionnaire completion. The questionnaire was then either administered by telephone, with verbal consent electronically documented by the interviewer and responses entered in real time into REDCap® (36% of the participants), or self-administered on the same platform through a secure form sent to participants by e-mail that included an electronic consent checkbox (64% of the participants).

**Figure 1.**
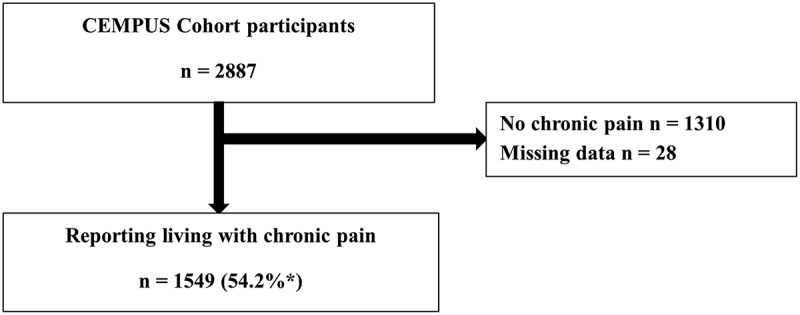
Flowchart for inclusion and exclusion of participants.

The convenience sample selected for the present study was composed of the 1549 participants of the CEMPUS Cohort who reported living with pain for at least 3 months (considered as chronic pain). A flowchart for inclusion and exclusion of participants is shown in Fig. 1.

*Valid percentage calculated after listwise deletion of cases with missing data.

The characteristics of our CEMPUS Cohort subsample are relatively comparable to those of probabilistic samples of people living with chronic pain regarding sex (64% of the females in our sample vs. 55–65% in other samples) and employment (45% of the employed participants vs. 38–67% in the other samples).^31–36^ Post-secondary education attainment was also comparable, with less than a 10 percentage-point difference (75% in our study vs. 68% in another sample).^36^ However, in terms of pain duration, people who had lived with chronic pain for at least 10 years were underrepresented in our study (28% vs. 46–47% in the two other probabilistic samples).^33,35^ Also, our sample consisted of slightly older individuals (the mean age was approximately 61 years old vs. 47–50 years old in other chronic pain samples).^33,34,36^ This highlights the importance of first stratifying prevalence results by age and pain duration in chronic pain studies using CEMPUS data, followed by the application of necessary multivariable adjustments to account for under- and over-representation.

The study protocol, along with the authorization for data access granted to all team members who worked with anonymized data, was approved by the human research ethics committee of CIUSSS-Estrie-CHUS, CEMPUS principal investigator’s institution (PF; Approval number: MP-31-2029-3172; Amendment form F1H-68285).

### Study variables

#### Food insecurity

The ten survey questions on food insecurity used in this study were based on the Institut national de santé publique du Québec (INSPQ)’s French adaptation^37^ of the adult Household Food Security Survey Module (HFSSM). This module was first included in the CCHS in 2005 and then in the annual Canadian Income Survey (CIS) in 2019.^38,39^ This is a well-validated instrument that allows Statistics Canada to monitor food insecurity across the country. While the full HFSSM includes 18 items (10 adult-referenced and 8 child-referenced),^14^ only the adult scale was used in the CEMPUS Cohort. This choice was justified given that not all participants had children in the household, that the study focused on individual-level outcomes, and to reduce respondent burden and limit missing data in the survey. Participants were asked about their household food security situation due to financial constraints over the last 12 months. Each question answered affirmatively was worth one point and the food insecurity score ranged from 0 to 10. According to this score, participants were divided into four categories intended for adult classification: food secure (no indication of income-related problems of food access; score of 0), marginally food insecure (worried about running out of food and/or limited food selection; score of 1), moderately food insecure (compromised in quality and/or quantity of food; score between 2 and 5 inclusive), and severely food insecure (missed meals, reduced food intake and, at the most extreme, went day(s) without food; scores ≥6).^40–42^ Combination of the last three categories allowed to create a binary variable for food insecurity (food secure/food insecure).

#### Chronic pain

Seven questions were asked about chronic pain. The first assessed the presence of chronic pain among all CEMPUS Cohort participants, using a 3-month duration cutoff as per the most recent recommendations.^1^ If a participant answered “yes” to the first question, further questions were asked: one question about pain frequency (continuous or occasional), one question about pain duration (number of years, categorized in our study into four groups: 3–11 months, 1–4 years, 5–9 years, and ≥10 years), and the four questions of the validated PROMIS Short-Form v1.1 – Pain Interference 4a (5-point Likert scales).^43^ This scale assesses the extent to which pain interfered with daily activities, housework, social activities, and household chores in the last 7 days, and allows the calculation of raw scores ranging from 4 to 20.^43^ Raw PROMIS Pain Interference scores were used to preserve interpretability within the sample. As T-scores are a linear transformation of raw scores, their use would not have altered the associations and was considered less relevant, given that the focus of the study was not to compare pain interference scores with external populations. The mean T-score was presented for descriptive purposes only, with the aim of facilitating interpretation for various readers.

#### Covariables (factors potentially associated with food insecurity)

The following variables were considered based on the scientific literature^5–9,13,16,25,26,41^ and clinical judgment: sociodemographic characteristics (age, sex, gender identity, occupation in the last 12 months, education level, total household before-tax income in 2023 (CAD), household type, presence of children in the household, country of birth), health profile and lifestyle variables (14-item Hospital Anxiety and Depression Scale (HADS),^44^ smoking status), and the above-mentioned chronic pain characteristics.

### Statistical analysis

Descriptive statistics (numbers and proportions for categorical variables; means, standard deviations, medians, and interquartile ranges for continuous variables) were used to portray the characteristics of the study sample and meet the first objective, i.e., to describe the prevalence of food insecurity among persons living with chronic pain. The prevalence of food insecurity was stratified across sex at birth, age, and pain duration groups. As per sex- and gender-based analysis recommendations,^45^ statistics should also be stratified across gender identity subgroups. However, in this study, given that the gender-diverse subgroup was small (n = 3), only sex at birth was used as a stratification variable or potential determinant. Gender identity was, however, described in Table 1. Age and pain duration were chosen as stratification variables because, as mentioned earlier, the CEMPUS cohort likely overrepresents older participants and underrepresents individuals with a pain duration of 10 years or more. Subgroup comparisons are presented for descriptive purposes only (no *p*-values were calculated), as associations between potential factors and outcomes were examined in the multivariable analyses described below, providing a more robust evaluation of independent relationships. To assess whether the trends observed in the CEMPUS cohort align with prior literature, the profile of food insecurity in our sample was also compared to that of CEMPUS participants without chronic pain and the Quebec population.

**Table 1.** Characteristics of the participants (n = 1549).

	n (%)
**Age (years), mean ± standard deviation**	61.1 ± 14.4
**Age (years)**	
18–24	14 (0.9)
25–34	66 (4.3)
35–44	162 (10.5)
45–54	216 (13.9)
55–64	344 (22.2)
≥65	747 (48.2)
**Sex**	
Female	967 (63.7)
Male	551 (36.3)
**Gender**	
Woman	965 (63.7)
Man	546 (36.1)
Gender diverse	3 (0.2)
**Occupation in the last 12 months**	
Employed	687 (44.5)
Retired	715 (46.3)
Non-employed (other than retired)	141 (9.1)
**Post-secondary education**	
Yes	1128 (74.5)
No	386 (25.5)
**Total household income in 2023**	
<30 000 CAD	205 (14.8)
30 000 CAD-69 999 CAD	465 (33.5)
70 000 CAD-149 999 CAD	525 (37.8)
150 000 CAD and more	194 (14.0)
**Household type**	
Couple (with or without children)	967 (87.4)
Single-parent family	85 (7.7)
Other	54 (4.9)
**Presence of children in the household**	
Yes	331 (21.4)
No	1217 (78.6)
**Born in Canada**	
Yes	1456 (95.9)
No	62 (4.1)
**Pain duration**	
3 months-11 months	241 (18.1)
1 year-4 years	505 (38.0)
5–9 years	206 (15.5)
≥10 years	378 (28.4)
**Pain pattern**	
Constant	622 (40.2)
Occasional	924 (59.8)
**Raw PROMIS pain interference score, mean ± standard deviation**	8.6 ± 4.1
**PROMIS pain interference T-score, mean ± standard deviation**	54.6 ± 0.2

As for the second objective, a multivariable logistic regression model was used to examine the association between factors potentially associated with food insecurity (dichotomous dependent variable). The following factors were included in the model: sociodemographic profile (sex, age, country of birth, household income in 2023, employment, post-secondary education, and presence of children in the household), health variables (anxiety (HADS-A score), depression/HADS-D score, and smoking status), and chronic pain characteristics (PROMIS score and pain duration). As our analysis aimed to identify factors potentially associated with food insecurity, and specifically to estimate the association between chronic-pain-related factors and food insecurity while accounting for potential confounders, covariables were selected *a priori* among relevant CEMPUS variables based on existing literature and clinical knowledge to represent key factors that may influence chronic pain or food insecurity. Following recommendations,^46^ this approach was preferred over criticized selection methods, such as relying on bivariate regression p-values or stepwise procedures.^46,47^ A parsimonious modeling strategy was adopted, given the relatively small number of individuals in the smallest outcome category (n = 154). Categories were combined when appropriate based on similarity in their association with the outcome (e.g., post-secondary vs. no post-secondary education). Consistent with common practices in logistic regression, the number of events in the smallest outcome category was used to inform the maximum number of dummy variables included (≈10 events per variable),^48^ corresponding to a model with no more than 15–16 dummy variables. Although age was categorized into 18–24, 25–34, 35–44, 45–54, 55–64, and ≥65 years to stratify prevalence estimates and ensure comparability with Statistics Canada data,^49^ it was included as a continuous variable in the multivariable model.

Crude and adjusted odds ratios (ORs) with 95% confidence intervals (CIs) were reported. The multivariable model quality was evaluated by confirming that CIs were not excessively wide, assessing multicollinearity (variance inflation factors < 5), and testing overall fit using the Hosmer–Lemeshow test (p > .05).^46,47^ Missing data were low for all variables included in the multivariable model (ranging from 0 to 2.3%), except for the household income (10.3%) and pain duration (14.1%). A sensitivity analysis was thus conducted to assess whether the imputation of missing values affected our conclusions. Missing values were handled using multiple imputation by fully conditional specification (FCS),^50^ generating five imputed datasets. The FCS algorithm was run for ten iterations to maximize the convergence of the imputations for both continuous and categorical variables. All analyses were performed using SPSS Statistics version 31® (IBM Corp, Armonk, NY).

## Results

Of the 2887 participants of the CEMPUS Cohort in Quebec, Canada, 1549 (54.2%) reported having chronic pain and therefore composed our study sample (Figure 1). The characteristics of the sample are presented in Table 1. Over two-thirds of the participants (70.4%) were at least 55 years old. In terms of sex and gender identity, 63.7% were assigned female sex at birth; 63.7% identified as women, 36.1% as men, and 0.2% as gender-diverse. The majority had post-secondary education (74.5%). Employed participants (44.5%) and retirees (46.3%) were present in similar proportions, and few were non-employed other than retired (9.1%). Many people had experienced chronic pain for between 1 and 4 years (38.0%). Over one in four participants (28.4%) had lived with chronic pain for at least 10 years. Fewer had lived with chronic pain for 3–11 months (18.1%) or for 5–9 years (15.5%).

Food insecurity variables among this study’s sample of people living with chronic pain (n = 1549) are shown in Table 2. Reference values for the CEMPUS cohort as a whole and the population of Quebec (according to Statistics Canada^51^) were also added to Table 2 for comparison purposes. In our study’s sample, the prevalence of food insecurity, all categories included, was 9.9%. When looking at the categories, 3.9% were marginally food insecure, 4.1% were moderately food insecure, and 2.0% were severely food insecure. The prevalence of food insecurity among females (10.0%) and males (10.3%) was comparable.

**Table 2.** Food insecurity variables among this study’s sample of people living with chronic pain, the CEMPUS Cohort and the population of Quebec.

Food insecurity variables	Chronic pain sample			Comparators		
	CEMPUS cohort – With chronic pain (n = 1549)	Females (sample)	Males (sample)	CEMPUS cohort – Total sample (n = 2887)	CEMPUS cohort – Without chronic pain (n = 1310)*	Quebec population**
Food insecurity status (binary) – n (%)						
Food secure	1395 (90.1)	868 (89.8)	496 (90.0)	2611 (90.4)	1189 (90.8)	(80.2)
Food insecure	154 (9.9)	99 (10.2)	55 (10.0)	276 (9.6)	121 (9.2)	(19.8)
Food insecurity subcategories – **n (%)**						
Marginally food insecure	60 (3.9)	36 (3.7)	24 (4.4)	114 (3.9)	56 (4.0)	(6.3)
Moderately food insecure	63 (4.1)	40 (4.1)	23 (4.2)	112 (3.9)	49 (3.7)	(9.8)
Severely food insecure	31 (2.0)	23 (2.4)	8 (1.5)	50 (1.7)	19 (1.5)	(3.7)
Moderately/severely food insecure – **n (%)**	94 (6.1)	63 (6.5)	31 (5.6)	162 (5.6)	68 (5.2)	(13.5)

Footnotes: SD: standard deviation; *No statistically significant differences were observed between CEMPUS participants with and without chronic pain for food insecurity variables. **Statistics from the 2023 CIS of Statistics Canada, measuring % of individuals aged 16+ living in food-secure and food-insecure households.^49^ Comparisons with those estimates based on the full 18-item HFSSM should be interpreted with caution, as the ten-item adult scale used in our study does not capture child-specific experiences and may underestimate household-level food insecurity.

**Figure 2.**
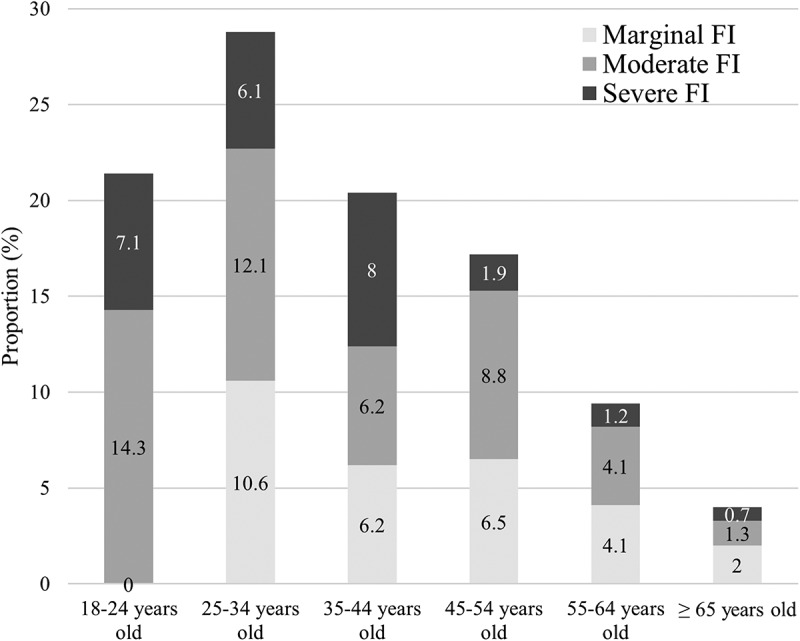
Prevalence (%) of food insecurity subcategories across age groups in this study’s sample.

Table 3 presents food insecurity prevalence by age group. Once again reference values from the literature^52^ were incorporated into the table for comparison. Figure 2 illustrates the prevalence (%) of food insecurity subcategories across age groups in this study’s sample. Food insecurity was less prevalent among older individuals. Food insecurity was reported by 27.5% of the adults aged 18–34, 18.5% of those aged 35–54, and 5.7% of the adults 55 and older. Prevalence of food insecurity was 11.9% among participants reporting pain for at least 10 years vs. 9.6% among those reporting pain for less than 10 years.

Footnotes: FI = Food insecurity.

**Table 3.** Food insecurity prevalence by age group among this study’s sample of people living with chronic pain (n = 1549) and the Quebec population.

Food insecurity status	Food secure -n (%)		Food insecure -n (%)	
**Age (years)**	Study sample	Quebec population*	Study sample	Quebec population*
18–24	11 (78.6)	(76.7)	3 (21.4)	(23.3)
25–34	47 (71.2)	(75.4)	19 (28.8)	(24.6)
35–44	129 (79.6)	(74.6)	33 (20.4)	(25.4)
45–54	179 (82.9)	(81.1)	37 (17.1)	(18.9)
55–64	312 (90.7)	(84.4)	32 (9.3)	(15.6)
≥65	717 (96.0)	(91.7)	30 (4.0)	(8.3)

*Statistics from the 2023 CIS of Statistics Canada, measuring % of individuals aged 16+ living in food-secure and food-insecure households.^49^ Comparisons with those estimates based on the full 18-item HFSSM should be interpreted with caution, as the 10-item adult scale used in our study does not capture child-specific experiences and may underestimate household-level food insecurity.

The estimates of the multivariable regression model created to analyze the factors associated with food insecurity among our sample of people living with chronic pain are shown in Table 4. According to the model, the following variables were associated with higher odds of experiencing food insecurity: (1) greater anxiety/higher HADS-A score (OR: 1.125, 95% CI: 1.051–1.205), (2) greater depression/higher HADS-D score (OR: 1.081, 95% CI: 1.004–1.164), 3) smoking (OR: 2.098, 95% CI: 1.234–3.566), (4) presence of children <18 years old in the household (OR: 1.927, 95% CI: 1.114–3.333), and (5) pain duration (1–4 years vs. 3–11 months OR: 2.086, 95% CI: 1.025–4.246). On the contrary, older age was associated with a decreased likelihood of food insecurity (OR: 0.952 95% CI: 0.934–0.969). Also, as compared to those with a household income <30 000 USD, participants with the household income between 30 000 USD and 69 999 USD (OR: 0.192 95% CI: 0.105–0.352), between 70 000 USD and 149 999 USD (OR: 0.073 95% CI: 0.036–0.148), or ≥150 000 USD (OR: 0.052 95% CI: 0.019–0.142) were less likely to be food insecure. Sex or pain interference was not associated with food insecurity independently of other covariables. The use of multiple imputation, which allowed us to gain statistical power, did not change the direction or statistical significance of the associations between the above-mentioned variables and food insecurity. However, it revealed an association not detected in the initial model: specifically, an association between employment and a lower likelihood of being in the food insecurity group (OR = 0.552, 95% CI: 0.334–0.915). HADS scores, smoking, presence of children <18 years old in the household, pain duration, pain interference, age, sex, and household income according to food insecurity severity are presented in Supplemental online material 1.

**Table 4.** Results from the multivariable regression model with crude and adjusted ORs and their 95% CIs.

Variables	Crude OR	95% CI		Adjusted OR	95% CI	
*Sociodemographic profile*						
Female sex (vs. male sex)	1.029	0.727	1.456	0.784	0.487	1.262
Age	0.948	0.938	0.959	**0.952**	**0.934**	**0.969**
Country of birth (Canada vs. other)	0.882	0.385	1.973	1.620	0.414	6.336
Household income (vs. <30 000 USD)						
Household income 30 000 USD-69 999 USD	0.256	0.216	0.303	**0.192**	**0.105**	**0.352**
Household income 70 000 USD-149 999 USD	0.148	0.122	0.178	**0.073**	**0.036**	**0.148**
Household income 150 000 USD and more	0.108	0.080	0.146	**0.052**	**0.019**	**0.142**
Employment (yes vs. no)*	0.648	0.463	0.906	0.734	0.422	1.276
Post-secondary education (yes vs. no)	0.675	0.471	0.967	0.992	0.579	1.699
Presence of children in the household	2.179	1.884	2.520	**1.927**	**1.114**	**3.333**
*Health variables*						
Anxiety (HADS-A score)	1.222	1.169	1.278	**1.125**	**1.051**	**1.205**
Depression (HADS-D score)	1.227	1.170	1.288	**1.081**	**1.004**	**1.164**
Current smoking status (smoking vs. nonsmoking)	3.632	3.086	4.275	**2.098**	**1.234**	**3.566**
*Chronic pain characteristics*						
PROMIS raw score	1.108	1.068	1.149	1.019	0.962	1.079
Pain duration (vs. 3 months-11 months)						
Pain duration 1 year-4 years	2.022	1.102	3.711	**2.086**	**1.025**	**4.246**
Pain duration 5 years-9 years	1.841	0.911	3.720	1.634	0.702	3.802
Pain duration 10 years or more	2.191	1.175	4.086	1.722	0.815	3.636

1178 participants out of 1549 were included in the final model. Bold indicates statistical significance in the multivariable model. *Statistical significance was reached after multiple imputation.

## Discussion

This study was conducted in Quebec, Canada, and aimed to describe the prevalence of food insecurity among people living with chronic pain and to identify associated factors. To our knowledge, this is the first study to examine food insecurity specifically in a chronic pain population. Overall, food insecurity affected approximately one in ten participants, but it was more frequent among younger individuals, affecting more than one in four. Some potentially modifiable associated factors, such as pain duration, anxiety, depression, smoking status, income, and employment, were identified.

### Prevalence of food insecurity

This study found that 9.9% of the people living with the chronic pain experienced food insecurity. While recent research has identified an association between food insecurity and chronic pain,^25,26^ prevalence had not been previously documented in that specific population. Contrary to our initial hypothesis, food insecurity was not found to be more prevalent among individuals without chronic pain or in the general population. In fact, the prevalence of food insecurity in our subsample of the CEMPUS Cohort was comparable to that observed in the whole CEMPUS Cohort (9.6%) or in participants without chronic pain (9.2%) but was about half that of Quebec’s general population (19.8% of the individuals aged 16+  and 19.0% of the households).^15,42^ Various factors should be considered when interpreting the prevalence of food insecurity in our sample, where the proportion of individuals reporting a household income below $30,000 (14.8%) is not far from that in the general population of Quebec (12.4%).^53^ First, comparisons with those estimates based on the full 18-item HFSSM should be interpreted with caution, as the ten-item adult scale used in our study does not capture child-specific experiences and may underestimate household-level food insecurity. However, age is likely to play an more important role, as differences between the prevalence observed in our study and that reported in the Quebec population appear less marked when age-stratified estimates are considered. Indeed, our sample is older than the general population of Quebec. The mean age is 42.7 years in Quebec,^54^ while it is 61.1 ± 14.4 years in our sample of people living with chronic pain. Older people experience more chronic pain^2,5,6,31,32^ and less food insecurity^13,16,41^ than younger people. Therefore, the older distribution of the population living with chronic pain could partly explain the lower prevalence of food insecurity in our sample. Our sample was also slightly older than other representative (probabilistic) samples of people living with chronic pain.^33,34,36^ This suggests that the overall prevalence of food insecurity among individuals living with chronic pain may be even higher than that estimated in the present study. Stratifying prevalence results by age group are likely a more valid way to present the estimates in our context. It revealed that food insecurity was substantially lower among older adults (21.4%, 28.8%, and 20.4% among participants aged 18–24, 25–34, and 35–44, respectively), which is consistent with the literature.^13,16,41^ This could be due to a more stable income, notably retirement pensions and a greater number of assets accumulated.^16,41^ Also, household composition could also explain the lower prevalence of food insecurity in our sample. Indeed, couples, with or without children, were markedly overrepresented in our sample (87.4% vs. 48.9% in the population of Quebec).^55^ Furthermore, most participants (78.6%) reported living in child-free households. Since couples, particularly those without at-home children have the lowest risk of food insecurity, whereas single-parent families face the highest risks,^16,41^ this could have contributed to the lower prevalence of food insecurity found in this study.

### Factors associated with food insecurity in our chronic pain sample

Subgroup comparisons suggested that older age was associated with lower odds of food insecurity, a finding confirmed in the multivariable analysis adjusting for household before-tax income. There was no indication of an association between sex and food insecurity in either our bivariable or multivariable analysis. This finding is consistent with recent Statistics Canada data indicating a similar frequency of food insecurity among females and males since 2018, with 26.0% of females and 25.0% of males affected in 2023.^52^ Although female sex alone was not identified as a significant risk factor, it might still influence food insecurity through gender-related differences in income levels. Recent Canadian research has demonstrated that families with a female major income earner, particularly female-led lone-parent families, had higher rates of food insecurity.^16,41^ This nuance was not examined in this study because gender roles were not assessed in the questionnaire. Further research on the interactions between sex, gender constructs, and food insecurity seems necessary.

Independently of other factors included in the multivariable model, higher income, and being employed were associated with decreased odds of being food insecure. Regarding income, our results are consistent with our findings in the general population.^13,16,41^ This was expected, as greater financial resources allow people to buy more varied and larger quantities of food, increasing the likelihood of being food secure. The association between employment and food insecurity, however, is more nuanced. It has been suggested that employment is associated with lower odds of food insecurity.^16^ However, individuals receiving public or private pensions may face a lower risk of food insecurity than those who are employed,^16,41^ and employment dimensions, such as income stability and working multiple jobs, may play an important role.^41,56^

The presence of children in the household remained associated with food insecurity even after adjusting for covariables, such as income. This association has been previously documented in the general Canadian population.^57^ Existent literature has also found that couples with children are at greater risk of food insecurity than those without, and children in general are at high risk of food insecurity.^16,41^ This may be explained by the substantial financial burden associated with raising children, which can limit the portion of the household budget allocated to food, despite higher household income levels.

Anxiety and depression were associated with an increased likelihood of being food insecure. This is also consistent with previous general population studies.^19,22–24^ This relationship could be bidirectional. The hardship and stress resulting from food insecurity may predispose individuals to develop psychological distress and mental illness. Anxiety and depression could also represent obstacles to professional opportunities and lead to socioeconomic disadvantage.

In our sample of individuals living with chronic pain, people who smoke were at greater risk of being food insecure than their nonsmoking peers. This is consistent with a recent review examining the intersection of food insecurity and tobacco use among the general population.^58^ This association is likely bidirectional. Tobacco-related expenditures can be a significant financial burden and therefore create or worsen the financial strain. On the other hand, smoking could be a coping mechanism in response to social and economic injustice. It has also been suggested that smokers living with food insecurity could use cigarettes to cope with hunger because nicotine reduces appetite.^58^

Pain duration of 1–4 years was associated with increased odds of being food insecure compared to pain duration of 3–11 months. No other study had specifically examined the relationship between chronic pain duration and food insecurity. Our hypothesis is that people who have lived with chronic pain for less than a year have not yet started to experience the extent of the negative financial impacts chronic pain can have, such as professional limitations and important health-related expenses. The association between longer pain duration (5–9 years and 10 years or more) and food insecurity was not statistically significant. This may suggest that, with time, people have adjusted to their condition and improved their coping strategies, making them less vulnerable. This is aligned with the findings of a previous study suggesting that the longer the duration of pain, the greater the pain relief reported by patients.^59^

### Recommendations

Research on topics that affect socioeconomically disadvantaged individuals is a step toward equitable access to healthcare for all and a Canadian priority.^60^ Our study allows us to formulate some concrete recommendations. Our findings demonstrate a substantial prevalence of food insecurity among individuals living with chronic pain, particularly among younger adults (e.g., more than one in four among those aged 25–34 years). This underscores the importance of measuring food insecurity in chronic pain studies designed to assess health equity. It would be important to develop data sources specifically designed for the study of food insecurity among persons living with chronic pain, including a broader range of clinical and social characteristics relevant to the pain and its management. In terms of clinical practice, some evidence suggests that screening for food insecurity and referring individuals to community resources may facilitate the identification of unmet needs and improve connections to support services.^61,62^ Although causal inferences cannot be made, our results suggest that food insecurity may represent an important consideration in chronic pain clinical practice and support further research on the potential relevance of screening approaches in this context.

### Strengths and limitations

Our study had several strengths. First, our sample size was substantial (n = 1549) and was similar to other probability samples of people living with chronic pain concerning their sex, employment, and education. Second, although the CEMPUS Cohort was not designed initially for chronic pain research, we used accepted definitions of chronic pain and food insecurity, as well as recognized and validated tools (e.g., Canadian Household Food Security Survey Module (HFSSM); PROMIS pain interference scale) to measure those variables and most others.

As for limitations, we acknowledge that this study was conducted in a single province and that geographic, policy, and social contexts play an important role in shaping both food insecurity and chronic pain. As such, our findings should be interpreted primarily within the Quebec context. While certain patterns observed may be consistent with those reported in other Canadian settings, differences in provincial policies, healthcare organization, and social determinants may limit direct generalizability. Nevertheless, given that Quebec shares several structural similarities with other Canadian provinces, our findings may offer relevant insights, although confirmation in other jurisdictions is warranted.

Our sample was older than other samples of people living with chronic pain,^33,34,36^ which opens to a selection bias. However, stratifying results by age groups allowed us to present prevalence estimates within each group, mitigating the impact of over-representation of older participants. Also, thanks to multivariable analyses, if age is overrepresented and associated with the outcome (e.g., food insecurity), including age as a covariate allows adjustment of estimates for other factors, enabling their associations to be assessed independently of the over-representation of specific age groups. People who had lived with chronic pain for 10 years or more seemed to be underrepresented in our study in comparison with other probability samples.^33,35^ However, another probability sample of people living with chronic pain had even lower rates in that category of pain duration, suggesting that our findings are not entirely inconsistent with existing evidence and may reflect normal variation across samples.^32^ The prevalence of food insecurity was also stratified across pain duration groups circumventing the selection bias (10 years or more vs. less than 10 years).

We would have found it interesting to develop a multivariable ordinal logistic model to identify factors associated with different levels of food insecurity (food secure vs. marginally food insecure vs. moderately food insecure vs. severely food insecure). However, our sample size did not allow for sufficiently powerful analyses. Nevertheless, we believe our study remains relevant, as all types of food insecurity, regardless of severity, are important to address. Also, some potentially relevant determinants of food insecurity, such as food assistance programs, alcohol use, housing conditions, social support, and income stability, were not included, either because these variables were not available in the CEMPUS cohort or for reasons of model parsimony, as described above. Future studies should examine these factors to better capture the complexity of food insecurity among individuals living with chronic pain.

Further work should explore differences in food insecurity between individuals with and without chronic pain while controlling for relevant covariates; potential interaction effects and mediators should also be examined. More in-depth analyses using an intersectional approach would also be highly valuable to better understand how overlapping social and health factors shape food insecurity in this population. Also, in our study, household-level food insecurity may be underestimated, particularly in households with children, as child-specific experiences were not captured using the ten-item adult HFSSM. Future research should consider using the full HFSSM to provide a more comprehensive assessment of household-level food insecurity. Finally, the cross-sectional design does not allow for causality to be established (e.g., potential bidirectionality between food insecurity and certain factors such as mental health). The temporal relationship between food insecurity and some of the identified associated variables should be further investigated using longitudinal and qualitative data. Future studies could also explore the links between social determinants of health, such as food insecurity and chronic pain development.

## Conclusion

To our knowledge, this is the first study to explore food insecurity and associated factors specifically among individuals living with chronic pain. In our sample of older adults living in the province of Quebec, Canada, food insecurity affects one in ten people living with chronic pain, with young adults disproportionately affected. Multiple modifiable factors were identified and may serve as potential targets for intervention. This study highlights the potential importance of integrating food insecurity screening in chronic pain care, as well as researching the impact of social determinants of health on chronic pain.

## Supplementary Material

Supplemental online material 1.pdf

## Data Availability

The CEMPUS Cohort is not readily available because participants did not initially provide consent to open data. Self-reported data are available from Dr. Paul Farand upon reasonable request and conditionally on a proper ethical approval for a secondary data analysis.
