# Supplementary material for: Prevalence Of Food Insecurity And Associated Factors Among Adults Living With Chronic Pain In Quebec, Canada: A Cross-Sectional Study
Source: Can J Pain. 2026 Jul 9;10(1):2680295. doi: 10.1080/24740527.2026.2680295 (PMC13353781; doi:10.1080/24740527.2026.2680295)
Supplement: Supplemental online material 1.pdf [file UCJP_A_2680295_SM6810.pdf]

Supplemental online material 1 – Sample characteristics according to food insecurity severity.

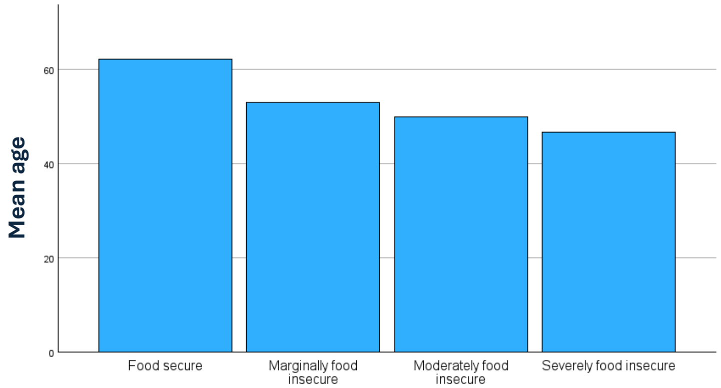

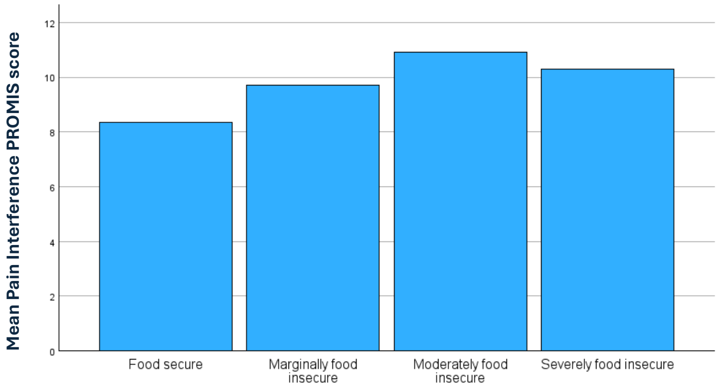

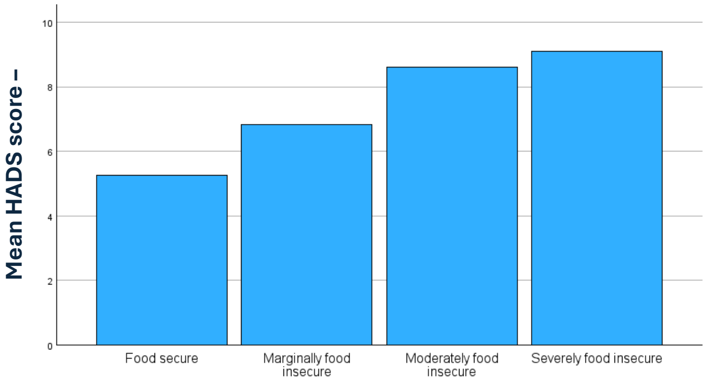

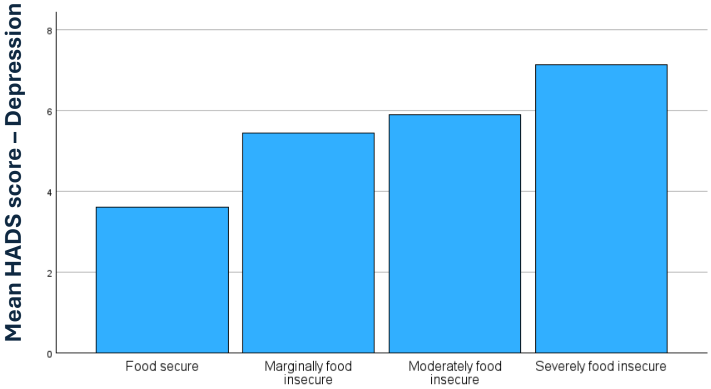

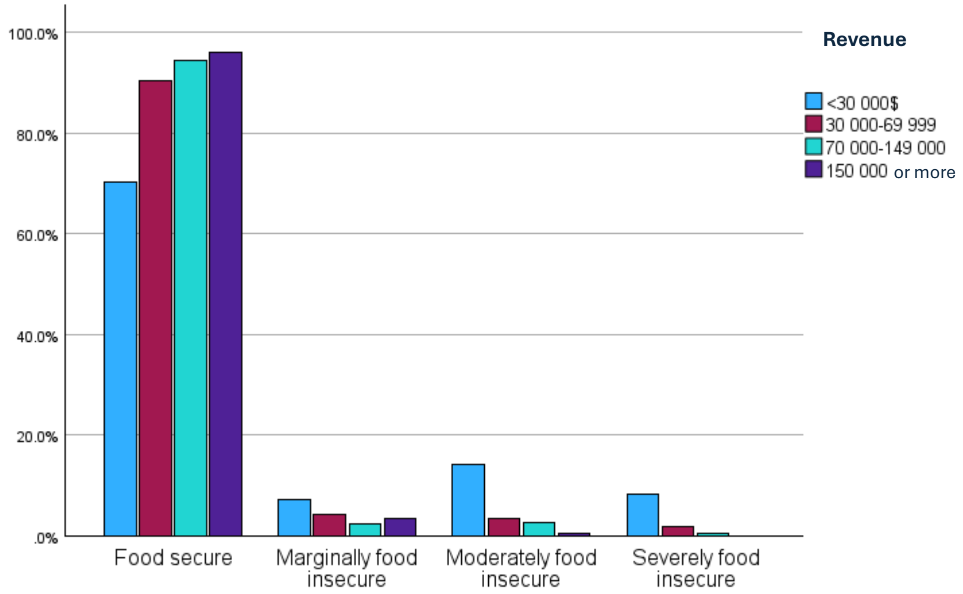

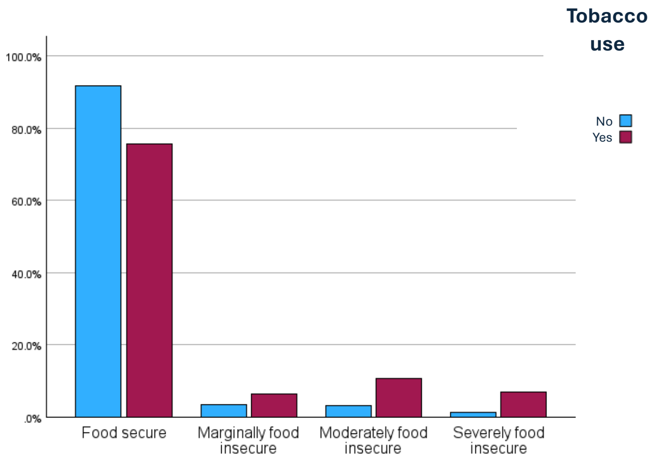
Tobacco use
No
Yes
100.0%
80.0%
60.0%
40.0%
20.0%
.0%
Food secure
Marginally food insecure
Moderately food insecure
Severely food insecure

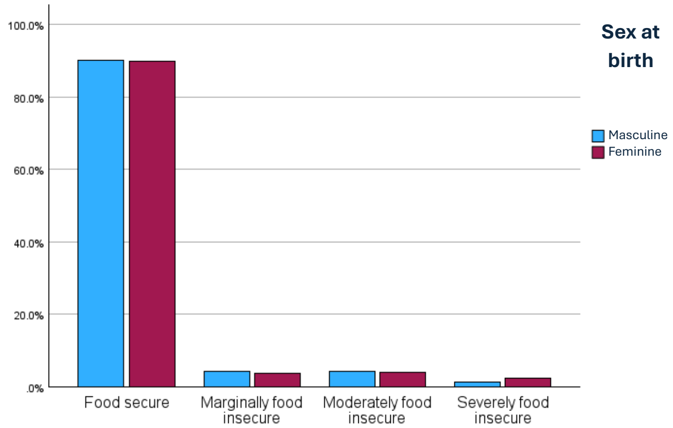
Sex at birth
Masculine
Feminine
100.0%
80.0%
60.0%
40.0%
20.0%
.0%
Food secure
Marginally food insecure
Moderately food insecure
Severely food insecure

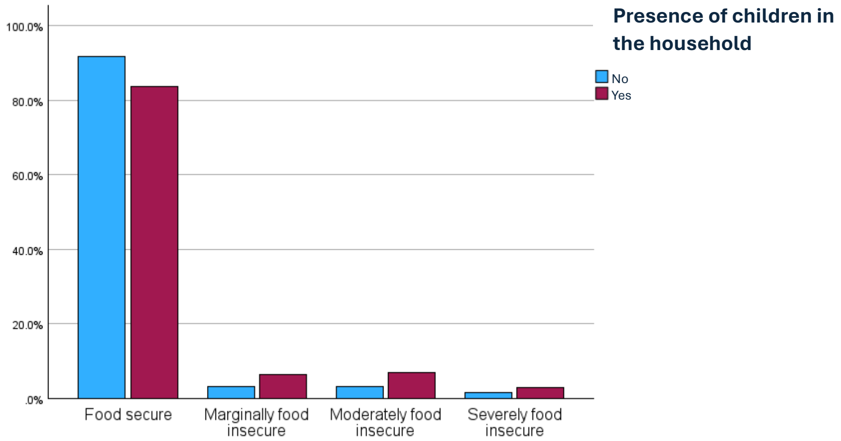
Presence of children in the household
No
Yes
100.0%
80.0%
60.0%
40.0%
20.0%
.0%
Food secure
Marginally food insecure
Moderately food insecure
Severely food insecure

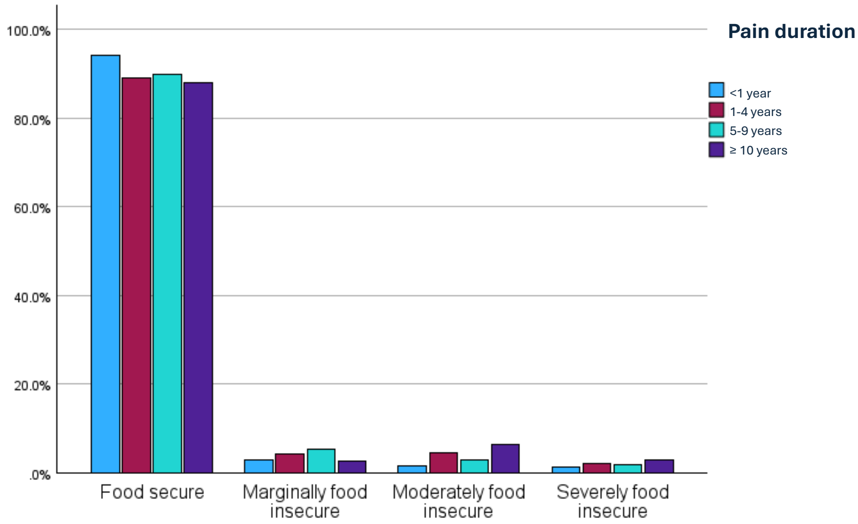
Pain duration
<1 year
1-4 years
5-9 years
≥ 10 years
100.0%
80.0%
60.0%
40.0%
20.0%
.0%
Food secure
Marginally food insecure
Moderately food insecure
Severely food insecure
